# Early developmental trajectories associated with different styles and intensities of ESDM-based community intervention

**DOI:** 10.1186/s11689-026-09698-w

**Published:** 2026-05-29

**Authors:** Liliana Ruta, Elisa Leonardi, Cristina Carrozza, Francesca I. Famà, Agrippina Campisi, Stefania Aiello, NEST Team, C Blandino, I Boccafoschi, M Boncoddo, M Bruschetta, I Crimi, F Di Bella, G Lazzaro, S Martines, A Orsina, M.G. Rosano, Flavia Marino, Alfia Ruggieri, Sabrina Baieli, Daniela Mangiapane, Gaetano Vivona, Sebastiano Marciante, Gaspare Cusimano, Antonio Narzisi, Filippo Muratori, Antonella Gagliano, Costanza Colombi, Gennaro Tartarisco, Marilina Mastrogiuseppe, Sally J. Rogers, Michael V. Lombardo, Giovanni Pioggia

**Affiliations:** 1 Institute for Biomedical Research and Innovation (CNR-IRIB), National Research Council of Italy, Istituto Marino Di Mortelle, Via Vincenzo Leanza, Snc, Messina, 98164 Italy; 2 University of Trieste, Trieste, Italy; 3 Centre for Autism Spectrum Disorders, Child Psychiatry Unit, Provincial Health Service of Catania, Catania, Italy; 4 Autism Centre, Child Psychiatry Unit, Provincial Health Service of Trapani, Trapani, Italy; 5 IRCCS Stella Maris Foundation, Pisa, Italy; 6 Child and Adolescent Neuropsychiatry, Department of Medicine and Surgery, “Kore” University of Enna, Enna, Italy; 7 Davis, Department of Psychiatry, University of California, MIND Institute, Sacramento, CA USA; 8 Laboratory for Autism and Neurodevelopmental Disorders, Center for Neuroscience and Cognitive Systems @UniTn, Istituto Italiano Di Tecnologia, Rovereto, Italy

**Keywords:** Early intervention, Autism, ESDM, TAU, Community setting, Developmental trajectories

## Abstract

**Background:**

The Early Start Denver Model (ESDM) is a naturalistic developmental behavioral intervention (NDBI) widely used to support early development in young autistic children. This study examines early developmental trajectories associated with different styles and intensities of ESDM-based intervention compared with community Therapy as Usual (TAU) over a 6-month period. It also explores predictors of individual language development based on the intervention style.

**Methods:**

A total of 112 autistic children participated in the study and were assessed longitudinally while receiving either higher (6 h a week) or lower (3 h a week) intensity ESDM, or TAU at higher (6 h a week) intensity (*N* = 29, 32, and 51 participants in each group, respectively).

**Results:**

The primary findings show that children receiving higher-intensity ESDM exhibited steeper developmental trajectories than children receiving TAU in all the developmental areas such as Language, Personal-Social skills, Performance, Eye-Hand coordination and the General score. Notably, lower-intensity ESDM was associated with steeper developmental gains in individual general development, language, personal social skills, and performance when compared to TAU at double the intensity. Additionally, secondary results indicate that language developmental trajectories are influenced by different factors in the ESDM and TAU groups, with social domain and adaptive behaviors predicting language progress in the ESDM group and baseline cognitive skills predicting language development in the TAU group.

**Conclusions:**

This study provides observational evidence that different intervention models and intensities may be associated with different short-term developmental trajectories in community settings, particularly where only limited weekly intervention hours are feasible. These findings may help inform service planning in low-resource contexts, while requiring replication in more controlled designs.

**Trial registration:**

Clinical Trial ID: NCT06494605.

**Supplementary Information:**

The online version contains supplementary material available at 10.1186/s11689-026-09698-w.

## Background

Autism is a neurodevelopmental condition characterized by difficulties in social communication, restricted and repetitive behaviors, and atypical sensory sensitivity [2]. It is lifelong and heterogeneous, with symptom severity and developmental trajectories varying from early childhood [6].

In the past two decades, research has demonstrated that autism can be reliably diagnosed at increasingly younger ages, as early as 14 months for some individuals [19]. Early identification is crucial to enable access to intervention programs that can support development and optimize outcomes from the earliest stages [7, 32].

Several standardized early intervention (EI) approaches have been developed and tested, differing in intervention style. Some follow the principles of Early Intensive Behavioral Intervention (EIBI) [20] or developmental science [13], while others—known as Naturalistic Developmental Behavioral Interventions (NDBIs)—combine behavioral and developmental principles within a naturalistic and interactive framework [9, 26]. A recent meta-analysis confirmed the effectiveness of NDBIs across multiple outcomes, while also emphasizing the importance of rigorous study design [25].

Within the NDBI framework, the Early Start Denver Model (ESDM) is a comprehensive intervention aimed at supporting development across all domains in very young autistic children (from infancy to age four). The ESDM [21] integrates developmental and behavioral principles to promote learning through play, shared attention, and social interaction within natural routines. Across multiple studies in different countries, ESDM has demonstrated greater gains in language, developmental quotient (DQ), and adaptive behavior, along with reduced autism symptom severity, compared to community interventions [10].

In addition to intervention style, early intervention programs vary in intensity, ranging from high-intensity programs (15–25 h per week) to low-intensity programs (a few hours per week, often parent-delivered) [8, 12, 18, 24, 28]. A recent randomized controlled trial by [23] compared ABA and ESDM approaches, both including parent training, delivered at 15 or 25 h per week. This study, the first to objectively examine the effects of intervention hours and style on child outcomes, found no significant differences between approaches or intensities.

Implementing evidence-based practices in real-world contexts remains challenging due to limited financial resources, a shortage of trained professionals, and the organizational demands of serving many children and families [31]. Consequently, in most community settings worldwide, it is difficult for families to access more than 3–6 h per week of professional intervention.

For these reasons, we examined developmental trajectories associated with ESDM delivered in a community setting at two low intensities (3 and 6 h per week) compared with the community-based Therapy as Usual (TAU) condition at 6 h per week. Although this study does not test efficacy under randomized conditions, it provides observational evidence on how different intervention experiences are associated with distinct developmental trajectories in young autistic children. The main comparison focuses on the combined effects of intervention intensity and approach across these three conditions. While it is important to examine main effects of intervention style on individual developmental slopes, the novel aspect of this study is testing the interaction between approach and intensity by contrasting two ESDM groups (6 h vs. 3 h per week) with the TAU condition (6 h per week).

Based on previous evidence, we expected that children receiving ESDM—when delivered at the same number of weekly hours as TAU—would show steeper developmental trajectories. We also expected that within the ESDM group, a higher intervention intensity would be associated with steeper developmental trajectories. Finally, we hypothesized that even at half the weekly intensity, ESDM would yield greater developmental progress than TAU delivered at double the intensity. Secondary analyses examined whether individual clinical characteristics at baseline moderated language gains as a function of intervention style. These hypotheses concern associations observed in a naturalistic context rather than causal effects.

## Methods

### Participants and procedures

A total sample of *n* = 117 young autistic children (19% females) were enrolled in the study between January 2012 and January 2022. Children were not randomized to condition but rather were followed within the community interventions and intensities that they received in a naturalistic setting. Out of the total sample, *n* = 5 children (*n* = 2 in the ESDM-6 group, *n* = 1 in the ESDM-3 group and *n* = 2 in the TAU group) discontinued participation in the study by the third month (*n* = 4 children for family and parental work reasons, while one child had to drop out of the study for medical reasons) and were excluded from the analysis. Hence a final sample of *n* = 112 children was examined. A total of 61 children participated in the ESDM intervention, which was administered at two distinct low intensities. Among them, 29 children were part of the ESDM-6 group, receiving on average 6 h weekly, and 32 children were part of the ESDM-3 group, receiving on average 3 h weekly. Additionally, 51 children received non-specific community interventions, namely TAU. Chronological age was 29.17 (SD = 8) months in the ESDM-6 group, 27.96 (SD = 6.1) months in the ESDM-3 and 31.92 (SD = 4.9) months in the TAU group. All the families who took part in the study were recruited and the intervention was carried out in clinical and territorial services in the south of Italy (Sicily), thanks to an agreement between the Messina Unit of the National Research Council Institute for Biomedical Research and Innovation (CNR-IRIB), the Neuropsychiatry Unit of the University Polyclinic Hospital "G. Martino" in Messina and the provincial community services in Catania and Trapani. Part of the sample in the ESDM-6 group and the TAU group has been previously published in [5]. Inclusion criteria: (a) a diagnosis of autism spectrum disorder according to Diagnostic and Statistical Manual of Mental Disorders 5th [2] and 4th [1] editions made by a qualified child neuropsychiatrist in the context of a multidisciplinary team and supported by the ADOS-2, (b) Italian as the main language spoken at home. Exclusion criteria: (a) any other identifiable genetic condition associated with autism (e.g., Fragile X syndrome, Cornelia de Lange syndrome, Tuberous Sclerosis Complex, Rett syndrome, Angelman syndrome, Prader-Willi syndrome), (b) epileptic encephalopathy with onset in infancy, and (c) significant sensory or motor impairment (e.g., vision or hearing impairment, cerebral palsy). The study received ethical clearance by the ethics committee of CNR and the local health ethics committees, and all the caregivers signed an informed consent to participate in the study.

### Measures

All children were assessed at baseline, 3 months, and 6 months using standardized developmental measures administered by trained clinical psychologists. The Griffiths Mental Development Scales – Extended Revised (GMDS-ER) were used at all timepoints to assess general and domain-specific development. Baseline assessments also included the Vineland Adaptive Behavior Scales – Second Edition (VABS-II) and the Autism Diagnostic Observation Schedule – Second Edition (ADOS-2), which were used as baseline predictors rather than repeated outcome measures. Intervention providers were distinct from outcome assessors. Developmental assessments were conducted by trained clinical psychologists who were not involved in the intervention delivery. However, due to the community service setting, assessors were not systematically blinded to group allocation.

*The Griffiths Mental Development Scales-Extended Revised (GMDS-ER).* The GMDS-ER [14] is widely used by clinicians to assess children from birth to 96 months of age in all areas of development, including children with developmental disorders and autism (e.g., [4]. The scale provides a General Quotient (GQ) as well as scores in different developmental domains: (i) Locomotor: measures gross motor skills, including the ability to balance, coordinate, and control movements; (ii) Personal Social: assesses skills that contribute to independence and social development; (iii) Hearing and Language: evaluates receptive and expressive language; (iv) Eye and Hand Coordination: targets fine motor skills, manual dexterity, and visual tracking skills; (v) Performance: measures the child's visuospatial skills, work speed, and accuracy. The GMDS-ER allows estimating an age-equivalent score (AE) that expresses the chronological age for which a given level of skill is average or typical and a standardized developmental quotient (DQ) score (DQ = (AE/chronological age) *100), with a standard mean of 100 and SDs of 15.

*Vineland Adaptive Behavior Scales, Second Edition (VABS-II).* The VABS-II [27] is a psychometrically validated instrument administered via semi-structured parent interview to measure a child's adaptive behavior in daily life in four domains: communication, daily living skills, locomotor and socialization. Skill in each domain is quantified with standardized scores indicative of where the child stands relative to age-norms. Additionally, VABS-II also allows for a final standardized adaptive behavior composite score (ABC). In this study, we utilized VABS-II standardized domain and ABC scores at intervention start as predictive measures, and not as outcome measures.

*The Autism Diagnostic Observation Schedule-2 (ADOS-2).* The ADOS-2 is a semi-structured, standardized assessment of communication, social interaction, play, and restricted and repetitive behaviors [15]. The ADOS-2 allows the assessment and supports the diagnosis of autism across age, developmental level, and language skills. The ADOS-2 provides an empirically derived algorithm that combines scores from the Social Affect (SA) and Restricted and Repetitive Behaviors (RRB) domains as well as a calibrated symptom severity scores (CSS). In the ADOS-2, a Toddler module is developed for children below 30 months, to assess behaviors associated with an autism likelihood very early on. It was administered and scored by assessors trained to research standards. We employed ADOS-2 scores at intervention start as predictive measures, and not as outcome measures.

### Intervention procedures

All participants were recruited as part of a single, continuous research program coordinated by CNR-IRIB which served as the lead center throughout the study period. Participants in the TAU group were recruited from the same community services as those in the ESDM groups to ensure comparable settings and minimize cohort-related differences. The same assessment instruments and scoring manuals (GMDS-ER, ADOS-2, VABS-II) were used throughout the 2012–2022 period, ensuring methodological continuity and longitudinal comparability of all measurements.

The intervention team consisted of licensed professionals experienced in behavioral sciences and developmental psychology (including speech and language therapists, psychomotor therapists, educational specialists, and psychologists). In the ESDM group, the same certified ESDM trainer and supervisor (LR) oversaw all intervention sites, ensuring consistency and adherence to a standardized center protocol across the three collaborating sites—the main CNR-IRIB unit and two public child psychiatry services located in neighboring provinces within the same geographical area. Prior to the beginning of the intervention, trainees participated in both the introductory and advanced ESDM workshops on site, held by certified ESDM trainers, and achieved ESDM fidelity standards (mean = 0.86, SD = 0.04) within six months of starting their training. To maintain intervention fidelity, all therapists participated in monthly peer-review meetings (held either online or in person), during which video-recorded sessions were reviewed and fidelity checklists were scored and discussed. Formal fidelity assessments were conducted quarterly, confirming that all therapists consistently maintained fidelity levels equal to or above 80% [21].

### ESDM intervention

The ESDM [21] is grounded in developmental psychology and applied behavior analysis. It emphasizes learning through play, joint attention, and everyday social interactions within a positive affective relationship between the child and therapist. The ESDM targets all major developmental domains—communication, social interaction, cognition, motor, and adaptive skills—through individualized teaching embedded in natural routines. Each child’s goals are derived from the ESDM Curriculum Checklist, which identifies current developmental levels and next learning steps. Teaching strategies include modeling, prompting, and natural reinforcement to encourage spontaneous communication and flexible behavior. Sessions are highly interactive, combining child-led activities with therapist-guided opportunities for skill acquisition.

The ESDM intervention was delivered individually for a 6-month period. Parents were present in the therapy room but were not involved in specific parent coaching. Each child participating in the ESDM-6 group received on average 6 h of intervention a week (3 × 2-h sessions per week; mean = 6.48, SD = 0.86), while the ESDM-3 group received on average 3 h of intervention a week (3 × 1-h sessions per week; mean = 2.68, SD = 1.14). Children in the ESDM groups were enrolled in a comprehensive early intervention program delivered within community services, and participation in other structured or manualized interventions (e.g., ABA or other specific models) was not reported or documented during the study period.

### TAU intervention

The TAU intervention was delivered individually for a 6-month period and consisted of 6 h of intervention a week (3 × 2-h sessions per week; mean = 6.24, SD = 1.21). TAU consisted of community-based interventions provided by local services and representative of the average support offered in Italy. It included a combination of speech and language therapy, neuro-psychomotor intervention, occupational therapy. Although these community-based interventions may have incorporated general behavioral or developmental principles, they were not delivered according to any standardized manualized protocol, nor was treatment fidelity monitored. None of the professionals who carried out the TAU intervention had been certified in a specific approach including behavioral analysis or ESDM.

### Analysis plan

All statistical analyses and graphical visualization were implemented in *R* (version 4.2.1). To check for differences between intervention groups at intervention start, we conducted univariate ANOVAs on demographic variables (age), ADOS CSS, VABS-II ABC, communication, daily living skills, and socialization, and on the GMDS-ER General Quotient (GQ) and Developmental Quotients (DQs) across all GMDS-ER domains (locomotor, personal-social, hearing and language, eye–hand coordination, and performance). Sex distribution between the intervention groups was assessed using chi-square tests.

We modeled longitudinal trajectories using linear mixed-effects models (R, lme4: lmer). The dependent variables were age-equivalent (AE) scores for each GMDS-ER domain and the General score. Fixed effects included chronological age (CA, in months), group (ESDM-6, ESDM-3, TAU-6), and their CA × group interaction. The three-group structure (ESDM-6, ESDM-3, TAU-6) combines intervention type and intensity within a single factor; thus, the analysis does not represent a fully crossed intensity × intervention design. Instead, the models tested the interaction between chronological age and group (age × group) to evaluate whether developmental slopes (ΔAE/ΔCA) differed across these conditions. Baseline covariates were age at intervention start and baseline GMDS-ER General Quotient (GQ) where appropriate (e.g., when the corresponding domain baseline was not matched across groups). Random effects were specified as (CA | subject), allowing both random intercepts and slopes for age. Time was modeled as chronological age centered at each child’s baseline, preserving the exact timing of assessments and accommodating unequal spacing and missing intermediate visits. Given the naturalistic, non-randomized design, these analyses estimate associations between intervention experience and developmental trajectories rather than causal effects. We report β (AE-months per CA-month) with 95% CIs, evaluate fixed effects with ANOVA F-tests (containment degrees of freedom), and control multiplicity across domain-level tests using FDR (q < 0.05). When CA × group was significant, we probed it with pairwise group contrasts (adjusted for multiplicity) to identify which comparisons drove the interaction. For interpretability, we confirmed that alternative models using months since enrollment (0, ~ 3, ~ 6) yielded convergent results.

For secondary analyses, subject-specific Hearing-Language slopes were extracted from a *lmer* model in which Hearing-Language age-equivalent scores were modeled as the dependent variable, chronological age was included as a fixed effect, and subject-specific random intercepts and random slopes for age were specified. Subsequently, we applied a linear regression model, with the GMDS-ER Hearing-Language slopes as the dependent variable and the pre-intervention ADOS-2 SA and RRB scores, GMDS-ER GQ score, and VABS-II ABC as the predictor variables, separately for the ESDM and TAU groups.

## Results

### Demographic and clinical characteristics of the sample at intervention start

At baseline, there were significant group differences in age, GMDS-ER GQ and DQ in Hearing-Language, Personal-Social, Eye-Hand coordination, as well as VABS-II ABC and Communication. Post-hoc tests showed that children in the ESDM-3 group were younger than those in the TAU group, while the ESDM-6 and ESDM-3 groups had higher scores than the TAU group in the GMDS-ER and VABS scores. Descriptive statistics regarding demographic and clinical information from all the participants are reported in Table 1.

**Table 1:** Demographic and clinical characteristics of the sample at intervention start

Measure	ESDM-6 (*N* = 29)	ESDM-3 (*N* = 32)		TAU (*N* = 51)	*F*	*p value*	Pairwise—group comparisons
Sex (M/F)	27/2	23/9		41/10		.103	-
Age (months)	29.17 (8)	27.96 (6.1)		31.92 (4.9)	4.55	.013*	ESDM-6 = ESDM-3 & TAU; ESDM-3 < TAU
ADOS-2 CSS	6.25 (1.7)	6.18 (1.8)		6.00 (1.4)	0.44	.646	-
GMDS-ER domain							
GQ	67.69 (16.3)	63.72 (15.7)		56.02 (11.5)	7.44	<.001***	ESDM-6 = ESDM-3; ESDM-3 & ESDM-6 > TAU
Hearing-Language	50.66 (23.2)	42.03 (15.5)		29.58 (13.9)	14.8	<.001***	ESDM-6 = ESDM-3; ESDM-3 & ESDM-6 > TAU
Personal-Social	64.55 (20.8)	63.97 (19.4)		49.02 (16.6)	9.73	<.001***	ESDM-6 = ESDM-3; ESDM-3 & ESDM-6 > TAU
Performance	73.41 (23.4)	69.03 (27.1)		66.15 (17.2)	1.08	.345	-
Eye-Hand Coordination	69.34 (20.1)	64.31 (18.5)		57.27 (14.7)	5.79	.004**	ESDM-6 = ESDM-3; ESDM-6 > TAU
Locomotor	82.86 (21.1)	82.71 (18.9)		78.96 (16.7)	0.60	.551	-
VABS-II domain							
ABC	71.86 (10.9)	69.90 (9.9)		65.83 (8.9)	4.08	.020*	ESDM-6 = ESDM-3; ESDM-3 & ESDM-6 > TAU
Communication	69.18 (14.4)		66.29 (14.9)	60.08 (11.9)	5.05	.008**	ESDM-6 = ESDM-3; ESDM-3 & ESDM-6 > TAU
Daily living skills	74.14 (15.9)		72.29 (12.0)	68.67 (10.2)	2.01	.139	-
Socialization	66.78 (11.5)		69.95 (9.4)	63.49 (11.8)	2.86	.062	-

*ESDM-6* Early Start Denver Model at 6 h/week, *ESDM-3* Early Start Denver Model at 3 h/week, *TAU* Therapy as Usual at 6 h/week, *ADOS-2* Autism Diagnostic Observation Schedule-2, *CSS* Calibrated Severity Score, *GMDS-ER* Griffiths Mental Development Scales – Extended Revised, *GQ* General Quotient, *VABS-II* Vineland Adaptive Behavior Scales, Second Edition, *ABC* Adaptive Behavior Composite

Number of participants for ADOS-2: ESDM-6 = 28, ESDM-3 = 24, TAU = 16; for VABS-II: ESDM-6 = 28, ESDM-3 = 21, TAU = 48

Between-group comparisons were conducted using one-way ANOVA for continuous variables and Chi-square tests for categorical variables (Sex). For variables with significant omnibus effects, Bonferroni-corrected pairwise t-tests were used to determine group differences. Reported F values refer to omnibus ANOVA tests. Significance codes: *** *p* <.001, ** *p* <.01, * *p* <.05

### Primary results: effects of intervention style and intensity on individual developmental trajectories

**Fig. 1:**
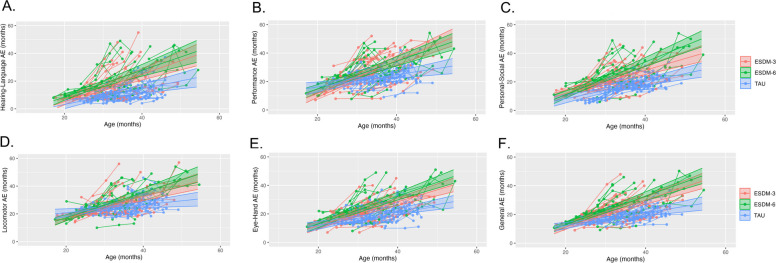
Effects of intervention style and intensity on developmental trajectories across chronological age. Group-level developmental trajectories are shown for the three intervention conditions: ESDM-6 (green), ESDM-3 (pink), and TAU (turquoise). Panels show developmental trajectories for the following domains: **A** Hearing-Language AE, **B** Performance AE, **C** Personal-Social AE, **D** Locomotor AE, **E** Eye-Hand AE, and **F** General AE. Lines represent estimated developmental slopes (ΔAE/ΔCA) derived from linear mixed-effects models controlling for baseline age and GMDS-ER GQ. Note: ESDM-6 = Early Start Denver Model at 6 h/week; ESDM-3 = Early Start Denver Model at 3 h/week; TAU = Therapy as Usual at 6 h/week; GMDS-ER = Griffiths Mental Development Scales – Extended Revised; AE = Age Equivalent

When we explored the effects of age and group on individual developmental trajectories, controlling for the effect of age at treatment start and the baseline GMDS-ER GQ, we found a main effect of age, group and their interaction in all the GMDS-ER developmental domains apart from the Locomotor domain where there was an effect of age and an interaction of age*group, but no main effect of group (Table 2, Fig. 1; see also Supplementary Fig. S1 for individual slope visualizations).

**Table 2:** Omnibus fixed effects from linear mixed-effects models on GMDS-ER developmental scores

GMDS-ER domain (AE)	*F*	*p value*
Hearing—language		
Effects of age	99.415	<.001
Effects of group	36.855	<.001
Effects of age at start	17.364	<.001
Effects of GQ at start	60.534	<.001
Age by group interaction	3.642	.028
Personal—social		
Effects of age	196.351	<.001
Effects of group	63.610	<.001
Effects of age at start	13.819	<.001
Effects of GQ at start	135.191	<.001
Age by group interaction	3.945	.021
Performance		
Effects of age	165.002	<.001
Effects of group	6.245	<.001
Effects of age at start	17.032	<.001
Age by group interaction	10.665	<.001
Eye-Hand coordination		
Effects of age	210.458	<.001
Effects of group	30.558	<.001
Effects of age at start	15.912	<.001
Effects of GQ at start	108.166	<.001
Age by group interaction	5.682	<.001
Locomotor		
Effects of age	114.706	<.001
Effects of group	2.827	.085
Effects of age at start	1.925	.168
Age by group interaction	9.717	<.001
GQ		
Effects of age	237.337	<.001
Effects of group	41.601	<.001
Effects of age at start	21.783	<.001
Effects of GQ at start	190.828	<.001
Age by group interaction	11.093	<.001

*GMDS-ER* Griffiths Mental and Developmental Scales Extended-Revised, *AE* Age Equivalent, *GQ* General Quotient

Table reports F-statistics and corresponding p-values for fixed effects (chronological age, intervention group, age at start, baseline GQ, and the age × group interaction) from the linear mixed-effects models. FDR adjusted *p*-values q < 0.05

Decomposing the main effect of age*group interaction in more detail for each pairwise-group comparison, we found that the ESDM-6 group displayed steeper intervention slopes compared to the TAU group on all the developmental domains. In turn, the ESDM-3 group showed intervention slopes comparable to the ESDM-6 group for Hearing-Language, Performance, Locomotor, Eye-Hand coordination and the General score, with a trend of significance for Personal-Social skills. Interestingly, compared to the TAU group, the ESDM-3 group, implemented at half the intensity, showed steeper developmental slopes in Hearing-Language, Personal-Social, Performance and the General score. Table 3 shows the pairwise-group comparisons.

**Table 3:** Pairwise comparisons on the GMDS-ER developmental scores

GMDS-ER domain (AE)	Pairwise differences of group		Age by group Interaction	
	*t*	*p value*	*t*	*p value*
Hearing—Language				
ESDM-6—ESDM-3	1.166	.476	0.964	.340
ESDM-6—TAU	4.426	<.001***	5.090	<.001***
ESDM-3—TAU	3.339	<.001***	3.775	<.001***
Personal-Social				
ESDM-6—ESDM-3	2.333	.056	1.767	.083
ESDM-6—TAU	5.933	<.001***	6.184	<.001***
ESDM-3—TAU	3.637	<.001***	4.736	<.001***
Performance				
ESDM-6—ESDM-3	0.412	.911	0.398	.692
ESDM-6—TAU	3.040	.039*	3.956	<.001***
ESDM-3—TAU	2.635	.025*	2.902	<.001***
Eye-Hand coordination				
ESDM-6—ESDM-3	1.924	<.001***	1.761	.084
ESDM-6—TAU	3.773	<.001***	3.699	<.001***
ESDM-3—TAU	1.829	.165	2.646	<.001***
Locomotor				
ESDM-6—ESDM-3	1.449	.320	1.112	.271
ESDM-6—TAU	3.543	<.001***	4.308	<.001***
ESDM-3—TAU	2.005	.174	2.491	.022*
GQ				
ESDM-6—ESDM-3	1.084	.526	0.926	.358
ESDM-6—TAU	4.316	<.001***	5.318	<.001***
ESDM-3—TAU	3.319	<.001***	4.240	<.001***

*GMDS-ER* Griffiths Mental and Developmental Scales Extended-Revised, *AE* Age Equivalent, *GQ* General Quotient

All analyses utilized linear mixed-effect models; FDR adjusted p-values q < 0.05. Significance codes: *** *p* <.001, ** *p* <.01, * *p* <.05

### Secondary results: clinical predictors of individual intervention trajectories on language development

**Fig. 2:**
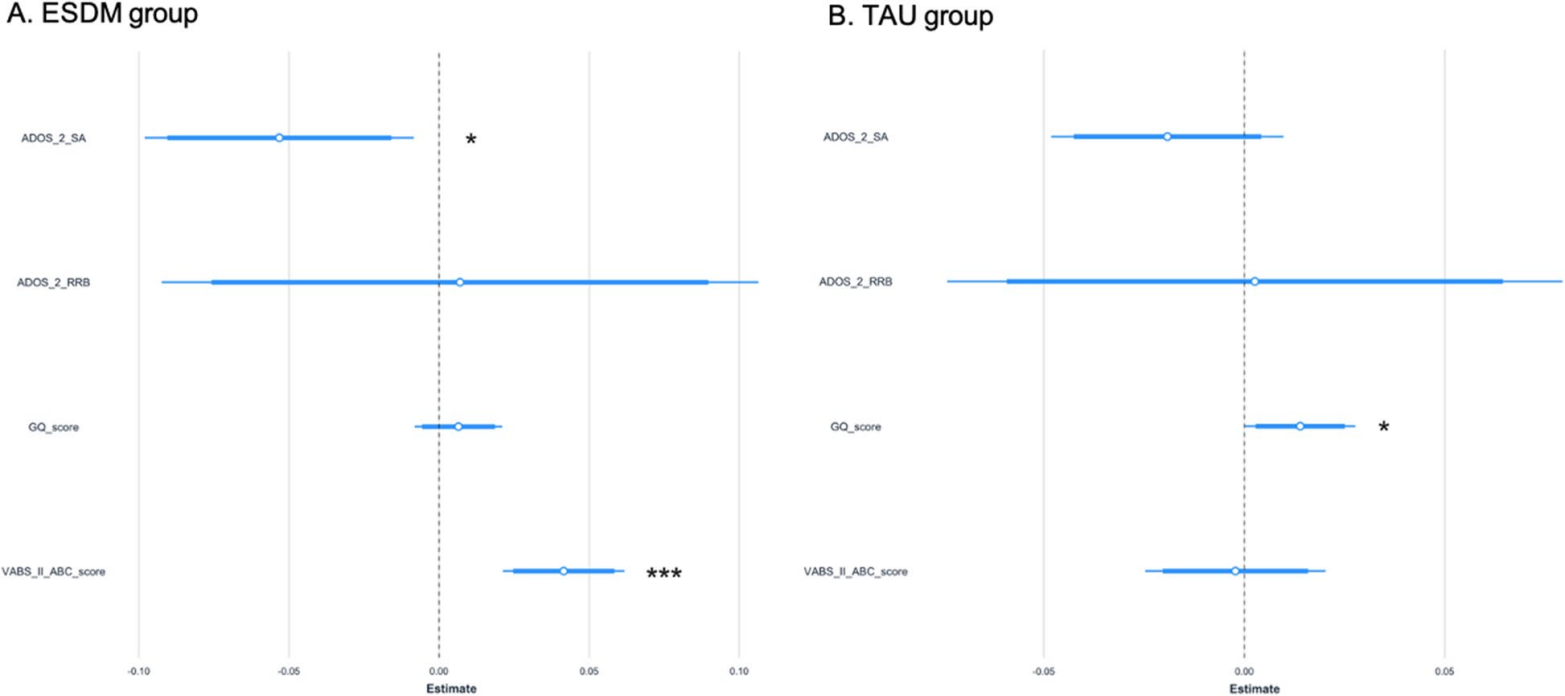
Predictors of language developmental slopes in the ESDM and TAU groups. Panel **A** Standardized regression coefficients (β) for pre-intervention predictors in the ESDM group. Panel **B** Standardized regression coefficients (β) for the same predictors in the TAU group. Positive coefficients indicate stronger association with steeper language developmental slopes over chronological age. Error bars represent ± 1 standard error. Note. ADOS-2 = Autism Diagnostic Observation Schedule-2; SA = Social Affect; RRB = Restricted Repetitive Behaviors; GMDS-ER = Griffiths Mental Development Scales – Extended Revised; GQ = General Quotient; VABS-II = Vineland Adaptive Behavior Scales II. *Significance levels:* *** *p* <.001; ** *p* <.01; * *p* <.05.*

Regression analysis showed a significant effect of the model in both the ESDM (*adjusted R*^*2*^ = 0.57, *F(4, 39)* = 15.15, *p* = 1.8e-7) and the TAU group (*adjusted R*^*2*^ = 0.82, *F(4, 8)* = 9.346, *p* = 4.1e-3), with different predictor variable's contribution in each group (see Table 4 and Fig. 2, Panel A and B, respectively). The graphical representation of these effects is shown in Fig. 2. In particular, in the ESDM group, the VABS-II ABC score and the ADOS-2 SA were significant predictors of Hearing-Language gains. Differently, in the TAU group, Hearing-Language gains were significantly predicted only by the GMDS-ER GQ (Fig. 2, Panel A and B, respectively).

**Table 4:** Clinical predictors of language slopes in the ESDM and TAU groups

Predictor	*Estimate*	*SE*	*β*	*p value*
ESDM				
Intercept	−1.823	0.753	−1.823	.020*
ADOS-2 SA	−0.053	0.022	-.053	.021*
ADOS-2 RRB	0.007	0.049	.007	.888
GMDS-ER GQ score	0.006	0.007	.006	.375
VABS-II ABC score	0.041	0.010	.041	<.001***
TAU				
Intercept	−0.369	0.584	-.369	.545
ADOS-2 SA	−0.019	0.012	-.019	.165
ADOS-2 RRB	0.003	0.033	.003	.939
GMDS-ER GQ score	0.014	0.006	.014	.048*
VABS-II ABC score	−0.002	0.010	-.002	.825

*ESDM* Early Start Denver Model, *TAU* Therapy as Usual, *ADOS-2* Autism Diagnostic Observation Schedule-2, *SA* Social Affect, *RRB* Restrictive and Repetitive Behaviors, *GMDS-ER* Griffiths Mental Development Scales – Extended Revised, *GQ* General Quotient, *VABS-II* Vineland Adaptive Behavior Scales, Second Edition, *ABC* Adaptive Behavior Composite

Significance codes: *** *p* <.001, ** *p* <.01, * *p* <.05

## Discussion

In this non-controlled community study, we examined developmental trajectories associated with delivering the ESDM intervention at two different low intensities (3 versus 6 h per week), compared with usual community therapy (TAU) delivered at 6 h per week, in a sample of 112 young autistic children. We observed that, at the same intensity of 6 h per week, children receiving the ESDM showed steeper developmental trajectories across all developmental areas than those receiving TAU. This pattern is consistent with previous literature characterizing the ESDM as a comprehensive early learning model that supports development in neurodivergent autistic children [5, 10]. Importantly, our findings extend this evidence to real-world community settings and to substantially lower weekly intensities than those typically examined in research trials.

Furthermore, ESDM-3 was associated with significantly greater developmental gains in Hearing-Language, Performance, Personal-Social domain and the General score, when compared to the growth made during TAU delivered at 6 h per week, twice the hours (and expense) of ESDM-3. To our knowledge, the only ESDM study directly comparing different intervention styles at different intensities is the randomized multi-site trial by [23], in which ESDM and ABA were delivered at 15 and 25 h per week, respectively, over a 12-month period. In that study, no significant differences were found between intervention styles or intensities on autism symptom severity, communication, language, or nonverbal ability. However, the study by Rogers and colleagues differs substantially from the present work. It was a 12-month randomized controlled trial conducted in a highly structured research environment and compared two manualized evidence-based interventions delivered at high intensity. In contrast, our study was carried out in community services and focused on developmental trajectories associated with ESDM delivered at two low intensities compared with a heterogeneous, non-standardized Therapy as Usual (TAU). Although our sample reflects a naturalistic convenience cohort, the patterns observed—particularly in early language development, a domain strongly associated with long-term outcomes in autism [16, 17]—provide informative insights into how different intervention experiences may relate to early developmental change in real-world settings. The observation that even 3 h per week of ESDM was associated with more accelerated language learning than twice as many hours of community intervention offers useful guidance for policy, particularly in contexts where resources are limited. When higher-intensity intervention is not feasible in most countries, it becomes essential to consider models that have demonstrated validity in supporting early social communication, language, and nonverbal development, and that can be delivered in formats compatible with what community services are structured to provide.

Beyond total intervention hours, the frequency and distribution of sessions across the week may also influence developmental trajectories. A schedule of three sessions per week, as implemented in all study groups, may promote more continuous engagement, support the consolidation of learning, and provide caregivers with more regular opportunities to observe and apply therapeutic strategies between sessions. This pattern of distributed practice may help facilitate generalization and sustain learning beyond the clinical setting, even when overall weekly intensity is low. Additionally, inter-individual differences in the autism phenotype are likely to contribute to variability in how children respond to different intervention styles. Taking into account that previous evidence supports the effectiveness of ESDM on language development [22, 29, 30] and considering our main results, which showed distinct patterns in language development between the ESDM and TAU groups, we explored whether specific behavioral characteristics at baseline were associated with individual trajectories of language growth following ESDM versus TAU therapy. Interestingly, we found that the SA domain of the ADOS-2, together with the VABS-II Composite score, at intervention start, significantly predicts greater language gains in the ESDM group, whereas the GMDS-ER GQ is the only significant predictor of greater language development in the TAU group. This result may suggest that, while higher levels of cognitive functioning and adaptive skills at intervention start positively influences child’s learning rate regardless of the style of support, children characterized by better social engagement and communication may find greater benefits in approaches that emphasize interactive naturalistic play, child initiative and shared enjoyment. These results are in line with a recent meta-analysis [3], suggesting that, within ESDM samples, early social-cognitive and language abilities at intake are associated with more favorable developmental trajectories. Our results are also in line with those of Godel and colleagues [11], who reported that, in a sample of children who received an ESDM support, baseline adaptive functioning combined with rapid developmental improvement during the first 6 months of intervention correlates with an optimal developmental outcome after 2 years of intervention.

### Limitations and strengths

Our study has several important limitations to acknowledge. First, because it was conducted in a community setting, it was not possible to randomize participants to the ESDM or TAU interventions or to different intensity levels and the associations reported cannot be interpreted as evidence of causal treatment effects. Instead, we adapted to the service structures in place, ensuring systematic and rigorous ESDM training and supervision within this framework. Additionally, certain clinical and demographic characteristics at the start of therapy were not fully balanced across groups. This imbalance may partly reflect the naturalistic enrollment procedures within community services and may therefore have influenced the findings. To address this concern, we conducted a sensitivity analysis in a restricted subsample with overlapping baseline characteristics, reported in the Supplementary Materials, which showed a pattern consistent with the main results. Nevertheless, children were allocated to either ESDM or TAU based on service waiting lists, and demographic and clinical variables at intervention start were either matched or statistically controlled in the analyses. It should also be noted that the TAU group does not represent a true control condition but rather a naturalistic comparison reflecting standard community-based services. Although TAU interventions were not manualized and differed somewhat across settings, they reflect the type of support typically available to families in public health systems. This heterogeneity may limit experimental precision but enhances ecological validity and the generalizability of the findings. Secondly, the assessors were not systematically blinded to group allocation, as complete masking was not feasible in the community service context. Nonetheless, intervention providers and outcome assessors were distinct, reducing the risk of measurement bias. Third, the intervention duration offered by the local services was short (six-month period), due to the high demand and long waiting lists. Therefore, no inferences on longer-term individual development trajectories could be done. However, having established three-month follow-ups made it possible to track early individual developmental trajectories on multiple time points. Last but not least, because group assignment followed a naturalistic service-based process rather than random allocation, unmeasured family or contextual factors (e.g., socio-economic status, caregiver engagement, prior familiarity with intervention models) cannot be entirely ruled out as potential influences on developmental outcomes.

Conducting the study in the face of logistic challenges and multi-level systemic resistance to its applicability allowed us the opportunity to initiate local cultural changes in the context of public early autism intervention, supporting a virtuous convergence of resources. We have demonstrated the feasibility of implementing manualized evidence-based intervention models at fidelity in the context of ordinary territory services activities, with the result of substantial benefits to children along with a major impact on the quality of community service performance.

### Clinical implications

Although it is important to bear in mind that our findings do not support the idea that very low intensities of intervention are optimal or even sufficient for long-term benefit for young children with autism, the main question that drove our research has important clinical implications for the correct allocation of resources in settings where they are truly scarce. Within this naturalistic community sample, lower-intensity ESDM was associated with more favorable trajectories in language, performance, and social development than higher-intensity non-standardized community interventions. These observations should be interpreted cautiously and do not establish treatment efficacy or causal superiority. In low-resource settings, they may nevertheless support consideration of therapist training and intervention quality as potentially important factors alongside intervention intensity. Therefore, based on our findings, it would be fundamental for policy makers and public autism intervention services in low resource settings where increasing the intensity of intervention is not sustainable, trying to invest in staff training to promote better developmental trajectories at a very early age, with a significant impact on early and long-term outcomes. However, further replication in studies with greater control is necessary to strengthen and generalize our findings. In addition, future research should explore, within community settings, how different low-intensity approaches—such as 1:1 intervention combined with parent-mediated strategies—can be integrated to further support early developmental trajectories in a sustainable and affordable way, with the broader goal of reaching all autistic children and families who require support worldwide.

## Conclusions

This study is unique in that it is the first to examine how different styles of intervention delivered at different low intensities within local community settings are associated with early developmental trajectories and short-term outcomes in young neurodivergent autistic children. Additionally, the study explored potential predictors of individual language outcomes as a function of intervention style. While the main results indicated that children receiving 6 h per week of ESDM showed steeper developmental trajectories than those receiving the same intensity of TAU, an important additional observation was that even 3 h per week of ESDM was associated with greater progress in language development, performance skills, social communication, and overall development compared with TAU delivered at double the weekly intensity.

In addition, secondary results suggest that language gains are differently predicted in the ESDM and TAU groups, in that the social domain and adaptive behaviors at intervention start predict language slopes in the ESDM group, while baseline cognitive skills predict language trajectories in the TAU group. This work may offer preliminary guidance for parents, clinicians, and policymakers regarding how different intervention styles and intensities are associated with developmental trajectories in young autistic children in low-resource community settings, while warranting confirmation in studies with stronger control of baseline group differences.

## Supplementary Information

Supplementary Material 1.

## Data Availability

The datasets used and/or analysed during the current study are available from the corresponding author on reasonable request.
