# Supplementary material for: Early developmental trajectories associated with different styles and intensities of ESDM-based community intervention
Source: J Neurodev Disord. 2026 May 29;18:46. doi: 10.1186/s11689-026-09698-w (PMC13412173; doi:10.1186/s11689-026-09698-w)
Supplement: Supplementary file 1 — Supplementary Material 1. [file 11689_2026_9698_MOESM1_ESM.docx]

### **Supplementary Materials**

**Supplementary Figure S1. Individual developmental slopes across chronological age (colored by slope magnitude).**

Individual model-estimated slopes are shown for each child, with colors reflecting slope magnitude (blue = lower slope; red = steeper slope). The color bar indicates ΔAE/ΔCA values, derived from linear mixed-effects models controlling for baseline age and GMDS-ER GQ. All models used chronological age as the continuous time variable.

This figure corresponds to the upper panel of the original Figure 1 in the main manuscript and is provided here to allow visualization of individual variability in developmental trajectories.

### **
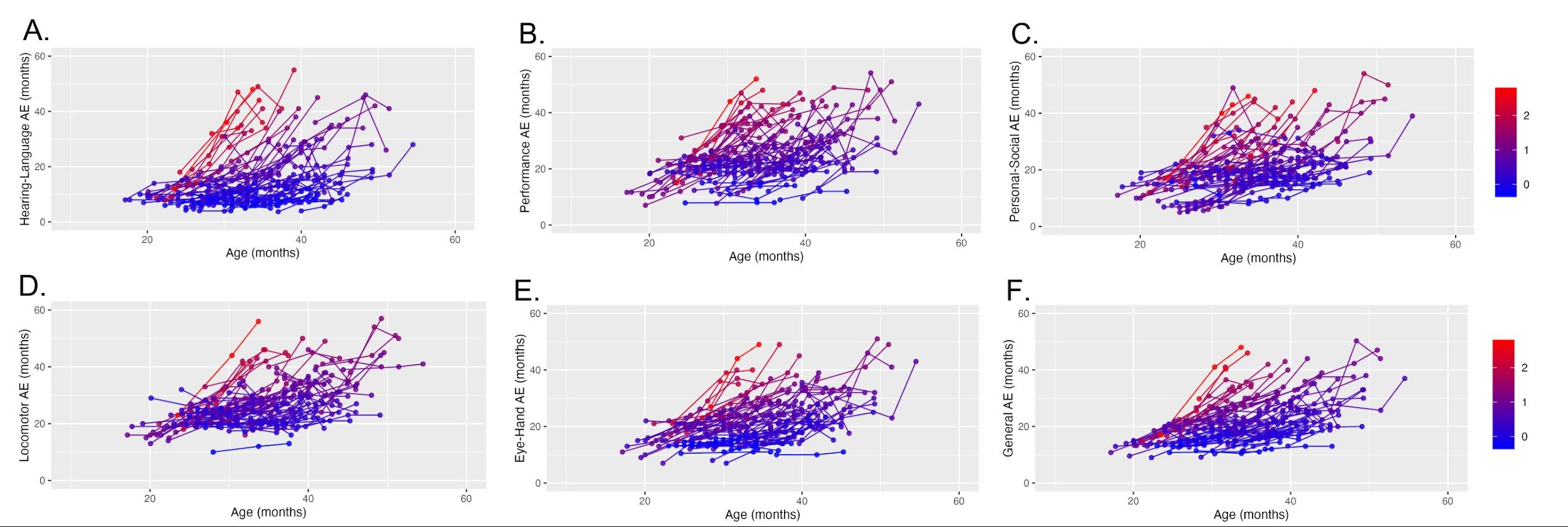
**

**Note.**ESDM-6 = Early Start Denver Model at 6 hours per week; ESDM-3 = Early Start Denver Model at 3 hours per week; TAU = Therapy as Usual at 6 hours per week; GMDS-ER = Griffiths Mental Development Scales – Extended Revised; AE = Age Equivalent.

**Sensitivity analysis in a restricted baseline-overlap subsample.** To assess the robustness of the main findings to baseline group imbalance, we conducted a sensitivity analysis in a restricted subsample including only participants within the overlap range for chronological age, baseline GMDS developmental quotient, used as the primary measure of developmental level, and Vineland composite score, used as an index of overall adaptive functioning.

Within this restricted subsample, the two ESDM subgroups were collapsed into a single ESDM group and compared with TAU (ESDM = 21; TAU = 48). Individual slopes derived from the longitudinal mixed-effects models were then compared between groups using Mann–Whitney U tests. The ESDM group showed significantly steeper slopes than TAU for Personal-Social (W = 805, p < .001), Performance (W = 790, p < .001), Locomotor (W = 768, p = .001), Eye-Hand Coordination development (W = 801, p < .001) and Total developmental level (W = 812, p < .001). Overall, these sensitivity analyses showed a pattern consistent with the main results, suggesting that the observed between-group differences were not solely attributable to baseline imbalance.
